# Lipoxin A4 protects primary spinal cord neurons from Erastin‐induced ferroptosis by activating the *Akt*/*Nrf2*/*HO‐1* signaling pathway

**DOI:** 10.1002/2211-5463.13203

**Published:** 2021-07-08

**Authors:** Na Wei, Tan Lu, Libin Yang, Yonghan Dong, Xiaotan Liu

**Affiliations:** ^1^ Department of Neurology Shanghai Tenth People's Hospital Chongming Branch Shanghai China; ^2^ Department of Orthopedics The First Affiliated Hospital of Xinxiang Medical University Weihui China; ^3^ Department of Orthopedics Xinxiang Central Hospital China; ^4^ Department of Orthopedics The Third Affiliated Hospital of Xinxiang Medical University China

**Keywords:** *Akt*/*Nrf2*/*HO‐1*, Erastin, ferroptosis, lipoxin A4, primary spinal cord neurons, spinal cord injury

## Abstract

Ferroptosis is an iron‐dependent programmed cell death, which participates in the pathogenesis of spinal cord injury (SCI). Our previous study has revealed that Lipoxin A4 (LXA4) exerts a protective role in SCI. Here, we investigated whether LXA4 can protect SCI through inhibiting neuronal ferroptosis. We treated primary spinal cord neurons with Erastin (ferroptosis activator) to induce ferroptosis. Erastin treatment reduced cell viability and enhanced cell death of primary spinal cord neurons, which was rescued by ferrostatin‐1 (ferroptosis inhibitor). Moreover, Erastin repressed glutathione peroxidase 4 (GPX4) expression and the levels of glutathione and cysteine in primary spinal cord neurons. Erastin also enhanced the expression of ferroptosis biomarkers (PTGS2 and ACSL4) and the levels of reactive oxygen species (ROS) in primary spinal cord neurons. The influence conferred by Erastin was effectively abolished by LXA4 treatment. Furthermore, LXA4 enhanced the protein expression of p‐AKT, nuclear factor (erythroid‐derived 2)‐like 2 (Nrf2) and haem‐oxygenase‐1 (HO‐1) in primary spinal cord neurons. LXA4‐mediated inhibition of ferroptosis of primary spinal cord neurons was prohibited by LY294002 (AKT inhibitor), brusatol (Nrf2 inhibitor) or zinc protoporphyrin (HO‐1 inhibitor). In conclusion, this work demonstrated that LXA4 exerted a neuroprotective effect in Erastin‐induced ferroptosis of primary spinal cord neurons by activating the *Akt*/*Nrf2*/*HO‐1* signaling pathway. Thus, this work provides novel insights into the mechanisms of action of LXA4 in ferroptosis of primary spinal cord neurons and indicates that LXA4 may be a potential therapeutic agent for SCI.

## Graphical abstract

Ferroptosis is closely associated with the pathogenesis of spinal cord injury. Erastin induced ferroptosis of primary spinal cord neurons, which was effectively abolished by Lipoxin A4 (LXA4) treatment. LXA4 exerted a neuroprotective effect in Erastin‐induced ferroptosis of primary spinal cord neurons by activating the Akt/nuclear factor (erythroid‐derived 2)‐like 2/haem‐oxygenase‐1 signaling pathway. Thus, LXA4 may be a potential therapeutic agent for spinal cord injury.

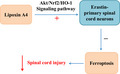

**Abbreviations**
BT: brusatol
CCK8: Cell Counting Kit‐8
Fer‐1: ferrostatin‐1
GPX4: glutathione peroxidase 4
GSH: glutathione
*HO‐1*
: haem‐oxygenase‐1
LXA4: Lipoxin A4
*Nrf2*
: nuclear factor (erythroid‐derived 2)‐like 2
ROS: reactive oxygen species
SCI: spinal cord injury
SD: standard deviation
WB: western blot
ZnPP: zinc protoporphyrin

Spinal cord injury (SCI) is a common traumatic neurological injury in orthopedics with a high rate of disability and fatality. SCI leads to a decrease or complete loss of motor and sensory functions below the injured spinal cord segment. The recovery of nerve function after SCI has always been a worldwide problem. SCI has devastating consequences for the physical, social, financial, and vocational well‐being of patients [1]. There is currently no effective treatment for SCI. The bottleneck of SCI treatment is to control the death of nerves and other cells. Researchers have discovered a new way of programmed cell death, iron‐dependent nonapoptotic cell death, ferroptosis [2]. The small molecule Erastin induces ferroptosis by inhibiting cystine–glutamate antiporter (System x_c_^−^) and inactivating glutathione peroxidase 4 (GPX4) [3]. This method of cell death is a form of iron‐dependent cell oxidative damage, which is mainly manifested by excessive lipid peroxidation and reduced lipid peroxidation removal ability. Ferroptosis is different from apoptosis, necroptosis, autophagy and other reported cell death methods [4].

Iron overload and lipid peroxidation have a vital role in SCI by inducing ferroptosis. Although iron is necessary for normal nerve function, iron overload aggravates secondary damage after nerve injury. Local hemorrhage in the acute phase of SCI leads to a significant increase of iron levels in the injured area, and iron overload further increases the accumulation of reactive oxygen species (ROS) [5]. *In vivo* study has demonstrated that irons play a very important role in the early stage of secondary SCI and enhance production of hydroxyl radicals [6]. The study of Koszyca *et al*. [7] also has confirmed that the expression of ferritin is highly expressed in SCI tissues, and ferritin expression is positively correlated with the severity of SCI. Therefore, intervention in the ferroptosis pathway is a new strategy for SCI treatment. Ferroptosis inhibitor, ferrostatin‐1 (Fer‐1), and iron chelators, deferoxamine, all play a neuroprotective effect in SCI by intervening ferroptosis [8, 9].

Lipoxin A4 (LXA4) is an important anti‐inflammatory mediator, known as the ‘stop signal’ of inflammatory response, which can promote resolution of inflammation. Our previous study has found that LXA4 protects SCI by activating the *AKT*/nuclear factor (erythroid‐derived 2)‐like 2 (*Nrf2*)/haem‐oxygenase‐1 (*HO‐1*) signaling pathway [10]. *Nrf2* is a core transcription factor that regulates cell oxidative stress, and it exerts an antioxidant/antiapoptotic effect. Multiple studies have shown that *AKT* and *Nrf2*/*HO‐1* signaling pathways participate in the regulation of ferroptosis [11, 12]. At the same time, the activated *AKT* and *Nrf2* signaling pathways attenuate Erastin‐induced ferroptosis [13, 14]. Thus, this study attempted to investigate whether LXA4 can inhibit Erastin‐induced ferroptosis of spinal cord neuron by activating the *AKT*/*Nrf2*/*HO‐1* signaling pathway.

## Materials and methods

### Animals

Pregnant C57BL/6 mouse was purchased from Laboratory Animal Center of Xinxiang Medical University. Pregnant mouse was housed under specified pathogen‐free conditions with free access to food and water. The experiment was approved by the Ethics Committee of the First Affiliated Hospital of Xinxiang Medical University.

### Isolation of primary spinal cord neuron

Primary spinal cord neurons were isolated from pregnant mouse as previously reported [15]. In brief, pregnant mouse was anesthetized with CO_2_ and sacrificed by cervical dislocation at embryonic day 15. All embryos were separated from pregnant mouse under aseptic conditions. Under dissection microscope, each embryo was quickly killed by cervical dislocation, and the spinal cord was isolated. The membrane of the spinal cord and dorsal root ganglion was removed from the spinal cord applying microforceps. Subsequently, the spinal cord was quickly cut into small pieces (1 mm^3^) using ultrafine microscissors. The sliced spinal cord was digested with 0.25% trypsin at 37 °C and 5% CO_2_ for 25 min. Dulbecco's modified Eagle’s medium/Nutrient mixture F‐12 (Gibco, Middleton, WI, USA) medium containing 2% FBS (Gibco) was added into the sliced spinal cord to terminate the digestion. Cell suspension was obtained by filtration with a 100 µm cell strainer (Boster, Wuhan, China).

### Cell culture and treatment

Primary spinal cord neurons were cultured in Dulbecco's modified Eagle’s medium/nutrient mixture F‐12 medium supplemented with 10% FBS and 1% penicillin/streptomycin (Solarbio, Beijing, China) at 37 °C and 5% CO_2_. Primary spinal cord neurons were treated with different concentrations of Erastin (0, 1, 5, 10 μm, ferroptosis activator; Aladdin, Shanghai, China) with or without LXA4 (10, 50, 100, 200 nm; Cayman Chemical Company, Ann Arbor, MI, USA) for 24 h. After Erastin with or without LXA4 treatment, cell morphology of primary spinal cord neurons was observed under an optical microscope. Primary spinal cord neurons were treated with 1 μm Fer‐1 (ferroptosis inhibitor, 24 h; Aladdin), 10 μm LY294002 (*AKT* inhibitor, 1 h; Aladdin), 20 nm brusatol (BT; *Nrf2* inhibitor, 2 h; Aladdin) or 20 μm zinc protoporphyrin (ZnPP, *HO‐1* inhibitor, 2 h; Cayman Chemical Company). Primary spinal cord neurons treated with 0.1% DMSO (Aladdin) were used as control.

### Western blot

Total protein was extracted from primary spinal cord neurons using Total Protein Extraction Kit (Solarbio). BCA‐100 Protein Quantitative Analysis Kit (Biocolors, Shanghai, China) was used to estimate the concentration of proteins. Equivalent protein samples (25 μg) were separated by 10% SDS/PAGE. The separated samples were transferred onto nitrocellulose membrane (Merck Millipore, Billerica, MA, USA). The membranes were blocked with 5% nonfat milk at room temperature for 2 h and then incubated with the primary antibodies, PTGS2 (1 : 1000; Proteintech, Wuhan, China), GPX4 (1 : 1000; Proteintech), ACSL4 (1 : 3000; Proteintech), AKT (1 : 1000; Proteintech), p‐AKT (1 : 2000; Proteintech), Nrf2 (1 : 1000; Proteintech), HO‐1 (1 : 1000; Proteintech) or β‐actin (1 : 2000; Proteintech) overnight at 4 °C. After washed with Tris‐buffered saline Tween for several times, the membranes were incubated with horseradish peroxidase‐conjugated secondary antibody (1 : 5000; Proteintech) at room temperature for 1 h. The data were analyzed by ImageJ software (National Institutes of Health, Bethesda, MD, USA).

### Cell viability

Cell Counting Kit‐8 (CCK8) assay was performed to examine cell viability of primary spinal cord neurons. In brief, primary spinal cord neurons were seeded into a 96‐well plate at a density of 5000 cells per well and incubated at 37 °C and 5% CO_2_ for 24 h. After treatment, CCK8 reagent (Sigma, Saint Louis, MO, USA) was added into each well and incubated with neurons for 4 h. The absorbance value of each well was measured at 450 nm (*A*_450 nm_) using a microplate reader (Bio‐Rad, Hercules, CA, USA).

### ROS assay

ROS production of primary spinal cord neurons was detected using DCFH‐DA fluorescence probe (Molecular Probes, Eugene, OR, USA). Primary spinal cord neurons were seeded into a six‐well plate at a concentration of 1 × 10^6^ cells per well and incubated at 37 °C and 5% CO_2_ for 24 h. After that, neurons were washed with PBS and then stained with 10 μm DCFH‐DA for 40 min. Finally, fluorescence intensity of each sample was observed using a fluorescence microscope. All images are representative of three independent experiments.

### ELISA

The levels of glutathione (GSH) and cysteine in primary spinal cord neurons were estimated applying Mouse GSH ELISA Kit and Mouse cysteine ELISA Kit (Jianglai, Shanghai, China) as the protocol described. The absorbance was detected applying a microplate reader (Thermo Fisher Scientific, Waltham, MA, USA).

### Statistical analysis

All assays were performed three times. The data were presented as mean ± standard deviation (SD). One‐way ANOVA and Student's *t*‐test were performed to analyze the statistical difference using graphpad prism software (GraphPad Software, La Jolla, CA, USA). *P* < 0.05 was considered to be statistically significant.

## Results

### Erastin‐induced ferroptosis of primary spinal cord neurons

**Fig. 1:**
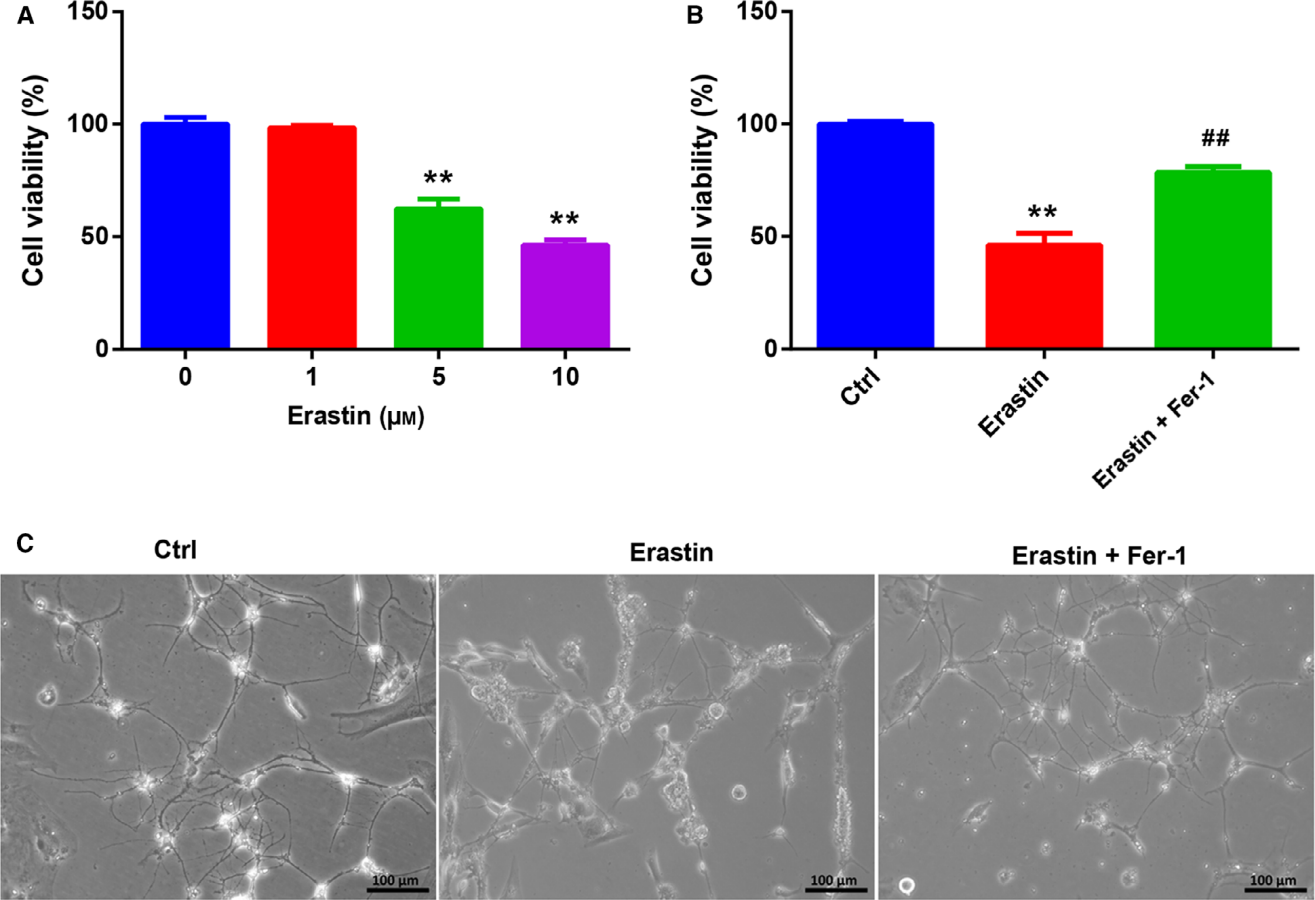
Erastin treatment promoted ferroptosis of primary spinal cord neurons. Primary neurons of the spinal cord were treated with different concentrations of Erastin (0, 1, 5, 10 μm) for 24 h. (A) CCK8 was performed to assess cell viability of primary spinal cord neurons. Student’s *t*‐test; the error bars indicate SD; *n* = 3. Primary spinal cord neurons were treated with Erastin (5 μm) with or without Fer‐1 (1 μm) for 24 h. Normal primary spinal cord neurons served as control. (B) CCK8 was performed to detect cell viability of primary spinal cord neurons. One‐way ANOVA; the error bars indicate SD; *n* = 3. (C) Cell morphology of primary spinal cord neurons was observed. Scale bar: 100 µm. **P < 0.01 vs. 0 or Ctrl group; ^##^ *P* < 0.01 vs. Erastin group.

We initially treated primary spinal cord neurons with Erastin to induce ferroptosis, and then we examined cell viability of primary spinal cord neurons. CCK8 assay results showed that Erastin at 5 and 10 μm significantly repressed cell viability of primary spinal cord neurons (Fig. 1A). We also found that Fer‐1 treatment rescued Erastin‐induced inhibition of cell viability of primary spinal cord neurons (Fig. 1B). Subsequently, we observed cell morphology of primary spinal cord neurons. Erastin treatment notably promoted cell death of primary spinal cord neurons, which was effectively abolished by Fer‐1 treatment (Fig. 1C).

### LXA4 inhibited Erastin‐induced ferroptosis of primary spinal cord neurons

**Fig. 2:**
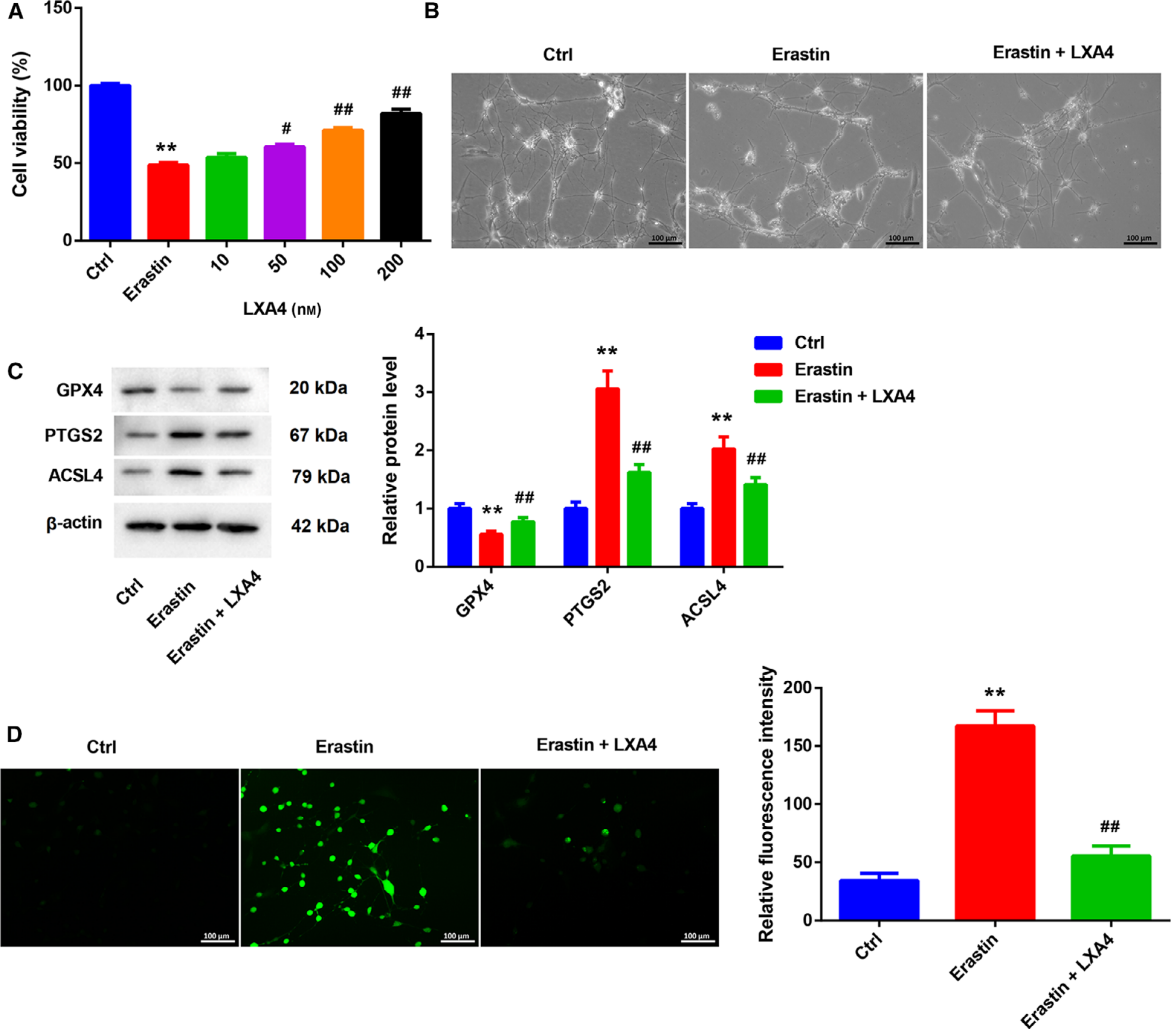
LXA4 inhibited Erastin‐induced ferroptosis of primary spinal cord neurons. Primary spinal cord neurons were treated with Erastin (5 μm) with or without different concentrations of LXA4 (10, 50, 100, 200 nm) for 24 h. Normal primary spinal cord neurons served as control. (A) CCK8 was performed to assess cell viability of primary spinal cord neurons. One‐way ANOVA; the error bars indicate SD; *n* = 3. Primary spinal cord neurons were treated with Erastin (5 μm) with or without LXA4 (100 nm) for 24 h. Normal primary spinal cord neurons served as control. (B) Cell morphology of primary spinal cord neurons was observed. Scale bars: 100 µm. (C) WB was performed to examine the expression of GPX4, PTGS2 and ACSL4 in the primary spinal cord neurons. One‐way ANOVA; the error bars indicate SD; *n* = 3. (D) DCFH‐DA probe was used to detect the levels of ROS in the primary spinal cord neurons. One‐way ANOVA; the error bars indicate SD; *n* = 3. Scale bars: 100 µm. **P < 0.01 vs. control (Ctrl) group; ^#^ *P* < 0.05, ^##^ *P* < 0.01 vs. Erastin group.

We further determined the biological role of LXA4 in Erastin‐induced ferroptosis of primary spinal cord neurons. The results obtained from CCK8 assay showed that Erastin treatment caused a decrease in cell viability of primary spinal cord neurons. However, LXA4 treatment (50, 100, 200 nm) enhanced cell viability of Erastin‐treated primary spinal cord neurons in a concentration‐dependent manner (Fig. 2A). Moreover, Erastin‐induced cell death of primary spinal cord neurons was attenuated by LXA4 treatment (Fig. 2B). We then estimated the influence of LXA4 on the expression of ferroptosis‐related proteins in primary spinal cord neurons. We found that Erastin caused a down‐regulation of GPX4 and led to an up‐regulation of PTGS2 and ACSL4 in the primary spinal cord neurons. The influence conferred by Erastin treatment was abolished by LXA4 treatment (Fig. 2C). Furthermore, the levels of GSH and cysteine in primary spinal cord neurons were examined through ELISA, showing that Erastin treatment significantly repressed the levels of GSH and cysteine in primary spinal cord neurons. LXA4 treatment rescued Erastin‐mediated inhibition of GSH and cysteine levels in primary spinal cord neurons (Fig. S1A,B). In addition, Erastin treatment enhanced ROS production in primary spinal cord neurons. LXA4 treatment reduced the levels of ROS in primary spinal cord neurons in the presence of Erastin (Fig. 2D).

### LXA4 inhibited Erastin‐induced ferroptosis of primary spinal cord neurons by activating the AKT/Nrf2/HO‐1 signaling pathway

**Fig. 3:**
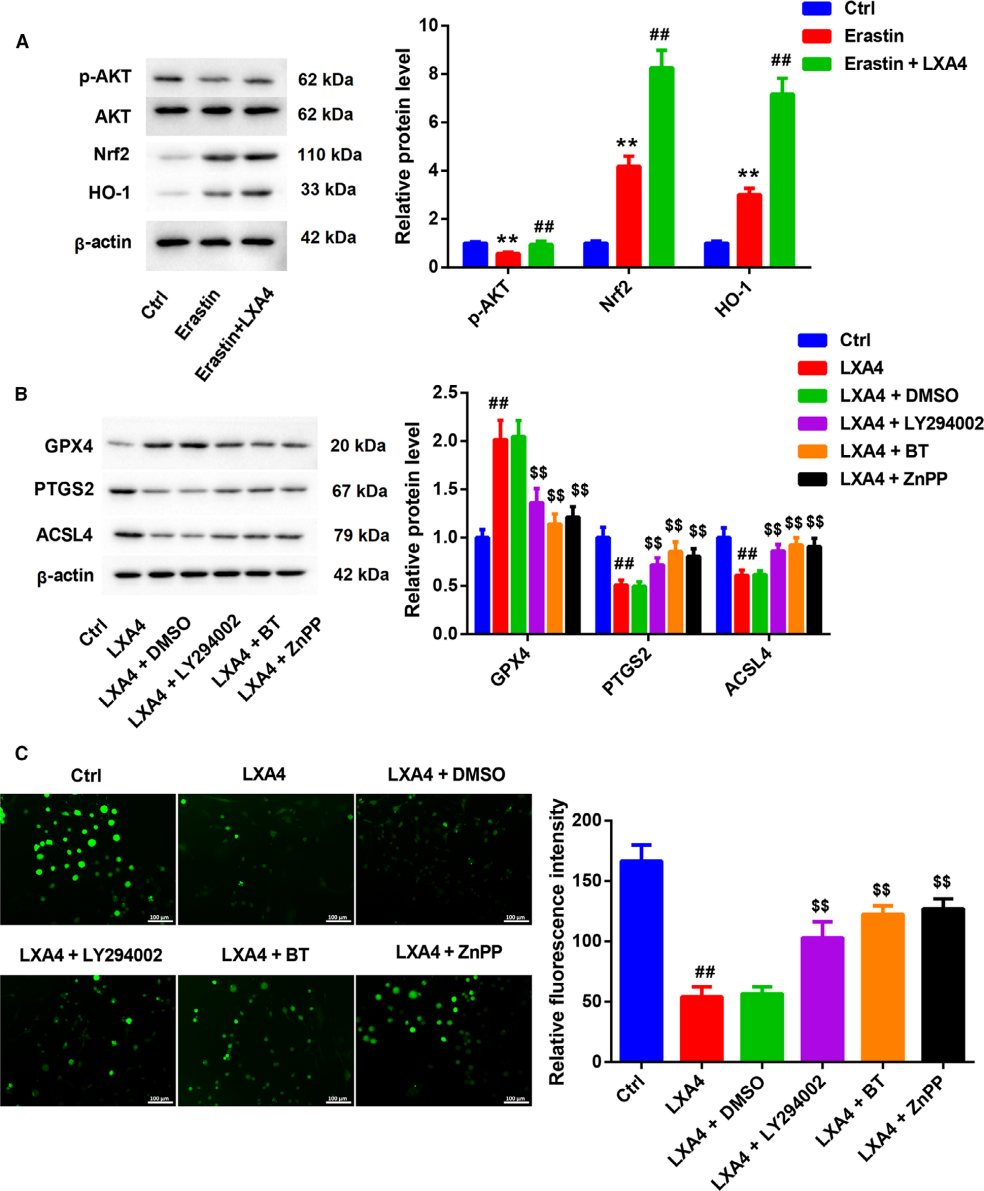
LXA4 inhibited Erastin‐induced ferroptosis of primary spinal cord neurons through the *AKT*/*Nrf2*/*HO‐1* signaling pathway. Primary spinal cord neurons were treated with Erastin (5 μm) with or without LXA4 (100 nm) for 24 h. Normal primary spinal cord neurons served as control. (A) WB was performed to examine the expression of p‐AKT, AKT, Nrf2 and HO‐1 in the primary spinal cord neurons. One‐way ANOVA; the error bars indicate SD; *n* = 3. Primary spinal cord neurons were treated with Erastin (5 μm) with or without LXA4 (100 nm) for 24 h, and then treated with LY294002 (10 μm) for 1 h, BT (20 nm) for 2 h or ZnPP (20 μm) for 2 h. Primary spinal cord neurons were treated with Erastin (5 μm) as control. (B) WB was performed to examine the expression of GPX4, PTGS2 and ACSL4 in the primary spinal cord neurons. One‐way ANOVA; the error bars indicate SD; *n* = 3. (C) DCFH‐DA probe was used to detect the levels of ROS in the primary spinal cord neurons. One‐way ANOVA; the error bars indicate SD; *n* = 3. Scale bars: 100 µm. **P < 0.01 vs. control (Ctrl) group; ^#^ *P* < 0.05, ^##^ *P* < 0.01 vs. Erastin group; ^$$^ *P* < 0.01 vs. LXA4 + DMSO group.

Finally, we determined whether LXA4 can inhibit Erastin‐induced ferroptosis of primary spinal cord neurons through the *AKT*/*Nrf2*/*HO‐1* signaling pathway. The expression of *AKT*/*Nrf2*/*HO‐1* signaling pathway‐related proteins was examined by western blot (WB). As shown in Fig. 3A, AKT protein expression had not changed in primary spinal cord neurons after the treatment of Erastin with or without LXA4. However, Erastin reduced p‐AKT expression and enhanced Nrf2 and HO‐1 expression in primary spinal cord neurons. LXA4 caused an up‐regulation of p‐AKT, Nrf2 and HO‐1 in the Erastin‐treated primary spinal cord neurons (Fig. 3A). We also found that LXA4 treatment enhanced GPX4 expression and repressed PTGS2 and ACSL4 expression in Erastin‐treated primary spinal cord neurons. However, GPX4 expression was inhibited by LY294002, BT or ZnPP in the Erastin combined with LXA4‐treated primary spinal cord neurons. LY294002, BT or ZnPP treatment led to an increase in the expression of PTGS2 and ACSL4 in primary spinal cord neurons in the presence of Erastin combined with LXA4 (Fig. 3B). Moreover, the levels of ROS were reduced in primary spinal cord neurons after Erastin combined with LXA4 treatment. LY294002, BT or ZnPP treatment rescued Erastin combined with LXA4‐mediated inhibition of ROS production in the primary spinal cord neurons (Fig. 3C).

## Discussion

LXA4 has been reported to exert anti‐inflammatory and analgesic effects in various diseases. For example, LXA4 treatment effectively represses microglial activation and inflammatory response, ultimately attenuating neuropathic pain in mouse and rat models of SCI [16]. LXA4 ameliorates neurological function and reduces cell apoptosis and oxidative stress in a rabbit model of ischemia–reperfusion‐induced SCI [17]. LXA4 exerts an anti‐inflammatory function in a rat model of noncompressive disk herniation by suppressing activation of NLRP3 inflammasome via the c‐Jun NH2‐terminal kinase 1/beclin‐1/class III phosphatidylinositol‐3‐kinase signaling pathway [18]. Ferroptosis is an iron‐dependent nonapoptotic cell death that plays a crucial role in SCI [2]. Ferroptosis is also an important means of neuron death, which plays an important role in central nervous degeneration and traumatic injury [19]. Deferoxamine can protect the primary cortical neurons from Erastin‐induced ferroptosis. The necroptosis inhibitor necrostain‐1 or the apoptosis inhibitor Z‐DEVD‐FMK has no effect on ferroptosis of primary cortical neurons caused by erastin [20]. Ferroptosis inhibitor SRS 16‐86 promotes functional recovery and neuronal survival, and represses inflammatory response by inhibiting ferroptosis in SCI rat models [21]. Here, we first confirmed the neuroprotective effect of LXA4 in SCI through regulating Erastin‐induced ferroptosis in primary spinal cord neurons. Erastin, as a ferroptosis activator, significantly induced ferroptosis of primary spinal cord neurons, which were rescued by Fer‐1, a ferroptosis inhibitor. However, LXA4 treatment effectively rescued Erastin‐mediated inhibition of cell viability of primary spinal cord neurons.

GPX4 is the main regulator of ferroptosis and maintains the balance of lipid metabolism in the microenvironment. Inhibition of GPX4 leads to lipid peroxidation, which induces the occurrence of ferroptosis [3, 22]. In brain trauma and secondary brain injury after intracerebral hemorrhage, down‐regulation of GPX4 leads to imbalance of the lipid oxidation microenvironment, ultimately inducing ferroptosis of neurons [23, 24]. As a limiting enzyme in the metabolism of arachidonic acid, PTGS2 is closely associated with ferroptosis [25]. ACSL4 functions as an essential component for ferroptosis execution and dictates ferroptosis sensitivity by shaping cellular lipid composition [26, 27]. Erastin inhibits the metabolism of cysteine to induce ferroptosis. System x_c_^−^ mediates the exchange of intracellular glutamate and extracellular cysteine [28]. Cystine is the raw material for the synthesis of GSH in the cells. Erastin inhibits the synthesis of GSH by inhibiting System x_c_^−^. GSH deficiency prevents cells from removing lipid peroxides, and thus causes protein and membrane damage and ferroptosis [29, 30]. In this study, Erastin treatment repressed GPX4 expression and enhanced PTGS2 and ACSL4 expression and ROS production in primary spinal cord neurons. The levels of GSH and cysteine in primary spinal cord neurons were repressed by Erastin treatment. The influence conferred by Erastin was abolished by LXA4 treatment. Thus, these data demonstrated that LXA4 inhibited Erastin‐induced ferroptosis of primary spinal cord neurons.

*PI3K*/*Akt* signaling has been reported to be involved in the pathological process of SCI. The study of Lv *et al*. [31] has revealed that miR‐21‐5p reduces apoptosis and inflammatory response of spinal cord tissues in a rat model of SCI by activating the *PI3K*/*AKT* signaling pathway. Neuron‐derived exosomal miR‐124‐3p exerts a neuroprotective effect in traumatically injured spinal cord by suppressing the activation of neurotoxic microglia and astrocytes through the *PI3K*/*AKT*/nuclear factor κB signaling pathway [32]. In our study, we found that Erastin repressed the expression of p‐AKT, whereas LXA4 caused an up‐regulation of p‐AKT in primary spinal cord neurons. Moreover, inhibition of AKT by LY294002 prohibited LXA4‐mediated inhibition of ferroptosis of primary spinal cord neurons. Thus, we speculated that LXA4 repressed ferroptosis of primary spinal cord neurons by activating the *AKT* signaling pathway.

The *Nrf2*/*HO‐1* signaling pathway is a key regulator in the pathophysiological response of oxidative stress, and it has become the main defense mechanism of neurons against oxidative stress [33]. Melatonin and Fer‐1 alleviate ferroptosis in high‐glucose‐treated MC3T3‐E1 cells or angiotensin II‐induced astrocytes through activation of the *Nrf2*/*HO‐1* signaling pathway [34, 35]. In this study, we found that Erastin treatment enhanced the expression of *Nrf2* and *HO‐1* in primary spinal cord neurons. LXA4 repressed ferroptosis of primary spinal cord neurons, which was rescued by BT‐mediated inhibition of *Nrf2* or ZnPP‐induced inhibition of *HO‐1*. Thus, these data demonstrated that in the presence of Erastin, up‐regulation of *Nrf2* and *HO‐1* may protect primary spinal cord neurons against oxidative stress. Moreover, LXA4 further activated the *Nrf2*/*HO‐1* signaling pathway to alleviate ferroptosis of primary spinal cord neurons.

In conclusion, these data demonstrated that LXA4 exerted a neuroprotective effect in Erastin‐induced ferroptosis of primary spinal cord neurons by activating the *Akt*/*Nrf2*/*HO‐1* signaling pathway. Thus, this work provides novel insights into the mechanisms of action of LXA4 in ferroptosis of primary spinal cord neurons and indicates that LXA4 may be a potential therapeutic agent for SCI.

## Conflict of interest

The authors declare no conflict of interest.

## Author contributions

NW conceived and designed the project; NW, TL, LY, YD and XL acquired the data; NW and TL analyzed and interpreted the data; TL wrote the paper. All authors read and approved the paper.

## Supporting information


**Fig. S1**. Lipoxin A4 (LXA4) inhibited Erastin‐induced ferroptosis of primary spinal cord neurons. Primary spinal cord neurons were treated with Erastin (5 μM) with or without LXA4 (100 nM) for 24 h. Normal primary spinal cord neurons served as control. The levels of glutathione (GSH) (A) and cysteine (B) in primary spinal cord neurons were examined through ELISA. Data were presented as mean ± SD. One‐way ANOVA; the error bars indicate SD; *N* = 3. **P < 0.01 vs. control (Ctrl) group; ^##^ *P* < 0.01 vs. Erastin group.

## Data Availability

The data that support the findings of this study are available from the corresponding author upon reasonable request.
