# Supplementary figures and images for: Lipoxin A4 protects primary spinal cord neurons from Erastin‐induced ferroptosis by activating the Akt/Nrf2/HO‐1 signaling pathway
Source: FEBS Open Bio. 2021 Jul 8;11(8):2118–26. doi: 10.1002/2211-5463.13203 (PMC8329788; doi:10.1002/2211-5463.13203)

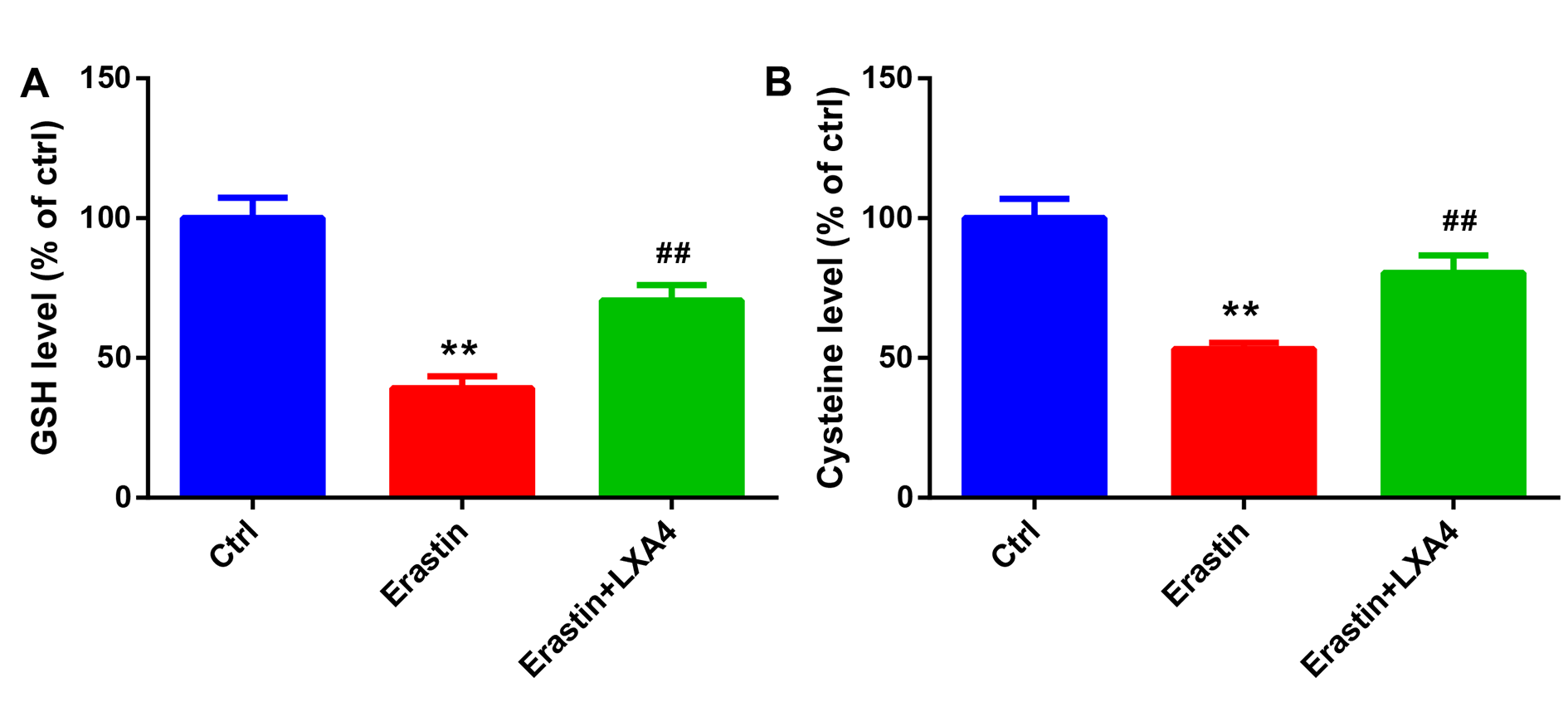

Supplement: Supplementary file 1 — Fig. S1. Lipoxin A4 (LXA4) inhibited Erastin‐induced ferroptosis of primary spinal cord neurons. Primary spinal cord neurons were treated with Erastin (5 μM) with or without LXA4 (100 nM) for 24 h. Normal primary spinal cord neurons served as control. The levels of glutathione (GSH) (A) and cysteine (B) in primary spinal cord neurons were examined through ELISA. Data were presented as mean ± SD. One‐way ANOVA; the error bars indicate SD; N = 3. **P < 0.01 vs. control (Ctrl) group; ## P < 0.01 vs. Erastin group. [file FEB4-11-2118-s001.tif]
